# Efficacy and safety of ticagrelor versus clopidogrel in patients with non-ST-elevation myocardial infarction in Taiwan

**DOI:** 10.1038/s41598-021-93712-9

**Published:** 2021-07-08

**Authors:** Chih-Kuo Lee, Tzung-Dau Wang, Hsiao-Ting Juang, Shu-Chen Chang, Heng-Yu Pan, Donna Shu-Han Lin, Chee-Jen Chang

**Affiliations:** 1 Division of Cardiology, Department of Internal Medicine, National Taiwan University Hospital Hsin-Chu Branch, Hsinchu, Taiwan; 2 College of Medicine, National Taiwan University, Taipei, Taiwan; 3 Cardiovascular Center and Division of Cardiology, Department of Internal Medicine, National Taiwan University Hospital, No. 7, Zhong-Shan South Road, Taipei City, 10002 Taiwan; 4 Clinical Informatics and Medical Statistics Research Center, Chang Gung University, Taoyüan, Taiwan; 5 Research Services Center for Health Information, Chang Gung University, Taoyüan, Taiwan; 6 Graduate Institute of Clinical Medical Sciences, Chang Gung University, Taoyüan, Taiwan; 7 Department of Cardiology, Chang Gung Memorial Hospital, Linkou, Taiwan; 8 Department of Biomedical Sciences, Chang Gung University, Taoyüan, Taiwan

**Keywords:** Cardiology, Cardiovascular diseases

## Abstract

The clinical efficacy of ticagrelor versus clopidogrel has not been replicated in East Asian populations. The pronounced bleeding risk with ticagrelor was of concern given the increased bleeding tendency in Asian populations. This study evaluated efficacy and safety of ticagrelor versus clopidogrel in patients with non-ST-elevation myocardial infarction (NSTEMI) in the entire Taiwan. We used the Taiwan National Health Insurance Research Database to identify 6203 patients aged ≥ 20 years with NSTEMI hospitalization and prescription of dual antiplatelets at discharge between January 2014 and December 2014. Cohorts of ticagrelor and clopidogrel were matched 1:1 based on propensity score matching to balance baseline covariates. The primary composite efficacy endpoints included death from any cause, non-fatal myocardial infarction, and non-fatal stroke. The secondary efficacy endpoints were the individual components. The primary safety endpoint was major bleeding requiring hospitalization. The incidence of primary efficacy endpoint was 20.3% in the ticagrelor users and 20.7% in the clopidogrel users (adjusted HR 0.94; 95% CI 0.73–1.22), with the median (interquartile range, IQR) follow-up period of 5.2 (2.3–8.5) months. The incidence of primary safety endpoint was 2.3% in the ticagrelor users and 3.2% in the clopidogrel users (adjusted HR 0.67; 95% CI 0.33–1.35). Regarding the secondary efficacy endpoint, patients treated with ticagrelor had significantly lower incidence of stroke (adjusted HR 0.44; 95% CI 0.21–0.94; *p* = 0.033). In this nationwide Taiwanese cohort of NSTEMI, treatment with ticagrelor after discharge, as compared to clopidogrel, had similar rates of ischemic composite events and major bleeding. Nevertheless, the median follow-up time was only 5.2 months, and the reduced stroke events with ticagrelor compared to clopidogrel needs further verification.

## Introduction

Current guidelines recommend ticagrelor over clopidogrel as part of standard therapy with aspirin in the treatment of patients with acute coronary syndrome (ACS), regardless of subtypes of myocardial infarction (MI)^1^. The recommendation is primarily based on the Platelet Inhibition and Patient Outcomes (PLATO) trial data in which ticagrelor reduced major adverse coronary events (MACE) versus clopidogrel. However, this superior efficacy was counterbalanced by increased bleeding and dyspnea^2^. Compared with patients with ST-elevation myocardial infarction (STEMI), patients with non-ST-elevation myocardial infarction (NSTEMI) appeared to have lower short-term mortality. While during longer-term follow-up, the mortality rates become comparable or even worse, likely due to older age and a greater prevalence of co-morbidities in NSTEMI patients^3, 4^. Currently, safety and efficacy of ticagrelor have not been studied sufficiently in patients with NSTEMI.

The consistency of efficacy and increased major bleeding risk of ticagrelor was observed in NSTEMI and Asian subgroups in the PLATO trial. The Asian population in the PLATO trial only represented 6% of all participants. The PHILO trial, which was designed to mirror PLATO and was conducted in Japan, South Korea and Taiwan, revealed numerically higher event rates of both primary safety and efficacy endpoints in ACS patients treated with ticagrelor compared with those assigned to the clopidogrel group^5^. Another randomized Ticagrelor Versus Clopidogrel in Asian/Korean Patients with ACS Intended for Invasive Management (TICAKOREA) trial targeting Asian populations also raised concerns regarding the significantly increased bleeding and numerically higher ischemic events with ticagrelor in Asian populations^6^. Patients in real world may differ in several aspects from those enrolled in clinical trials, including different baseline characteristics and lower likelihood of medication adherence^7^. The lack of adherence might have greater impact on ticagrelor, a reversible P2Y12 inhibitor, compared to other irreversible P2Y12 inhibitors. Therefore, whether the superior efficacy of ticagrelor in patients with NSTEMI, as observed in the PLATO trial, could be replicated in Asian populations is not certain. The increased risk of major bleeding with ticagrelor was another concern. According to the regulations of the Taiwan Food and Drug Administration (TFDA), drug licenses need to be renewed every five years. At the time of renewal, a real-world data (this study) was commissioned by the TFDA in 2017 to investigate the efficacy and safety of ticagrelor versus clopidogrel in the overall Taiwanese patients with NSTEMI by analyzing the nationwide claim-based dataset.

## Methods

### Study design

The license of Ticagrelor was approved in May 2012 by the TFDA, and Taiwan's National Health Insurance System has reimbursed Ticagrelor prescriptions for nine months after acute coronary syndrome since July 2013, according to the one year out-come of the Taiwan Acute Coronary Syndrome Full Spectrum Registry^8^. This nationwide population-based retrospective cohort study aimed to compare the efficacy and safety of ticagrelor versus clopidogrel in adult patients with newly developed events of NSTEMI in Taiwan with a maximum follow-up of 12-month. The Taiwan National Health Insurance Research Database in Health and Welfare Data Science Center is a nationwide database comprising anonymous eligibility and enrollment information as well as claims for visits, procedures, and prescription medications for more than 99% of the entire population (23 million) in Taiwan. In both the inpatient and outpatient databases, medical information including disease diagnosis, prescription medications, procedures, and surgery incurred during hospitalization or at an outpatient visit are documented. For processing by the National Health Insurance in Taiwan, it is mandatory for all health-care institutes to submit all diagnosis information using the International Classification of Disease-Clinical Modification, ninth revision (ICD-9-CM) together with service claims.

### Ethical approval

The study was approved and informed consent was waived by the institutional review board of National Taiwan University Hospital, Taipei, Taiwan (Joint Institutional Review Board number, 16-S-007-1). All patient information in the Taiwan National Health Insurance Research Database was anonymous and encrypted. All methods were performed in accordance with the relevant guidelines and regulations.

### Participants

**Figure 1:**
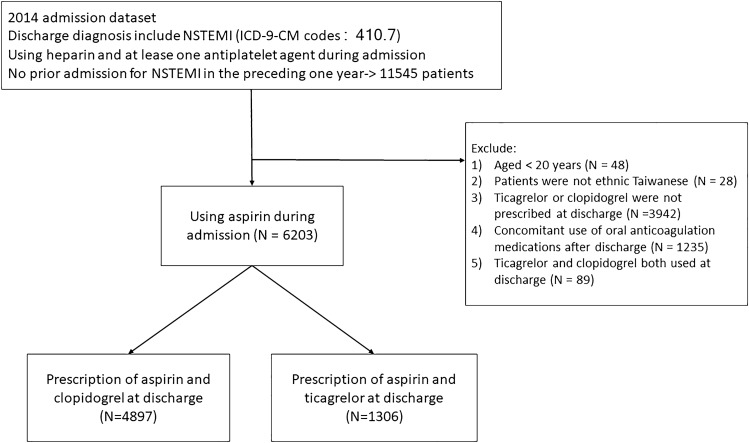
Participant selection.

We selected all adult patients (≥ 20 years) who were admitted to hospital for NSTEMI and discharged from 1 January 2014 to 31 December 2014. Diagnosis of NSTEMI was defined as hospitalization with a primary or secondary discharge diagnosis code of ICD-9-CM 410.7. Patients with previous NSTEMI hospitalization in the preceding one year were excluded. All patients should be treated with aspirin during hospitalization. They were divided into ticagrelor or clopidogrel group according to which drug was prescribed at discharge. Patients who were prescribed oral anticoagulation medications or more than two kinds of P2Y12 inhibitors at discharge were excluded (Fig. 1). For each patient, the comorbidities were extracted from both the inpatient and outpatient claim databases for 3 years before the index date. According to previous researches regarding validity of the ICD9-CM coding in Taiwan National Health Insurance Research Database, the accuracy of NSTEMI was 97.9% and the percentage of consistency in comorbidity diagnoses was 95.9%^9–11^. We obtained information on each patient at the index admission for NSTEMI, including age, gender, days of hospitalization, and the strategy of revascularization, including percutaneous coronary intervention (PCI) and coronary artery bypass grafting (CABG). The information regarding whether patients received intra-aortic balloon pump (IABP) supports were also collected. We ascertained medication use, both in-hospital and during follow-up, using the anatomic therapeutic chemical classification system, which included beta-blockers, calcium channel blockers, angiotensin-converting enzyme inhibitors (ACEi), angiotensin receptor blockers (ARB), statins, proton-pump inhibitors (PPIs) and glycoprotein IIb/IIIa inhibitors. The index date was defined as the discharge date for each group. The follow-up period was from the index date until the first episode of any study outcome or the end of the study period (30 December 2014), whichever came first.

### Treatment

In Taiwan, prasugrel was not available and ticagrelor and clopidogrel were both reimbursed for patients with NSTEMI for 9 months. After discharge, patients in the ticagrelor group received a dose of 90 mg twice a day and clopidogrel group received a dose of 75 mg daily. All patients received acetylsalicylic acid (aspirin) at a dose of 100 mg daily during NSTEMI admission and after discharge. Other medical treatments were also used based on the standard treatment regimen for patients with NSTEMI at physicians’ discretion.

### Study endpoints

The definition of primary efficacy outcome was composite of major cardiovascular events, including all-cause mortality, myocardial infarction, and stroke event. The secondary efficacy outcomes were the individual components of the primary efficacy outcome. We defined the primary safety outcome as major hemorrhage events which the patient needed to be hospitalized for further management. Major hemorrhage events included gastrointestinal (GI) bleeding and intracerebral hemorrhage. The definition of secondary safety outcomes were the individual components of the primary safety outcome.

### Statistical analysis

Demographic data are expressed as mean ± SD or percentage. In general, differences in proportions were analyzed using chi-squared test or Fisher’s exact test, and differences in parameters of continuous variables were analyzed using Student t-test. Because this is not a randomized controlled trial, propensity score analysis was performed to minimize any selection bias due to the differences in clinical characteristics between the groups. Propensity scores for the likelihood of receiving ticagrelor or clopidogrel were computed using multivariate logistic regression analysis, conditional on covariates including age, gender, cardiovascular risk factors, underlying diseases, in-hospital medications (beta-blockers, calcium channel blockers, ACEi, ARB, statins, proton-pump inhibitors and glycoprotein IIb/IIIa inhibitors), revascularization strategies and IABP supports. Then, using the Greedy’s Nearest Neighbor Matching, the propensity score was used to match ticagrelor or clopidogrel patients in a 1:1 ratio. Once a patient met the definition for exposure to ticagrelor or clopidogrel, he/she was considered exposed from that point forward, even if he/she discontinued therapy. Time-to-event Kaplan–Meier curves for each cohort were constructed for death from any cause, non-fatal MI, stroke, and a composite of primary efficacy endpoints and were compared using log-rank test. The estimates of relative risk, with 95% CI, were derived from Cox proportional hazards models, adjusted for potential confounding factors including medications and comorbidities, with matching analysis for propensity score matching. All analysis was performed using SAS version 9.4 (SAS Institute, Cary, NC, USA).

## Results

### Baseline characteristics

A total of 6203 patients with NSTEMI were identified. Of these, 4897 patients (78.9%) received aspirin and clopidogrel and 1306 patients (21.1%) received aspirin and ticagrelor at discharge (Fig. 1). Baseline clinical characteristics are listed in Table 1. Patients treated with aspirin and ticagrelor were younger than those who received aspirin and clopidogrel. There were significantly higher percentages of female gender and almost all comorbidities, except prior MI, peripheral vascular disease, dyslipidemia, and cancer, in the clopidogrel group than in the ticagrelor group. The use of guideline-directed medical therapy (beta-blocker, ACEi, and ARB) during hospitalization was significantly lower in the clopidogrel group. More patients in the clopidogrel group underwent CABG and fewer underwent percutaneous coronary intervention. After propensity score matching, 2606 patients (1303 in each group, 1:1) with NSTEMI were selected and baseline characteristics, comorbidities, revascularization strategies, and medications were well matched (Table 2).

**Table 1:** Baseline characteristics of subjects with non-ST elevation myocardial infarction.

	Clopidogrel	Ticagrelor	*p*-value
	(N = 4897)	(N = 1306)	
Age (mean ± SD)	69.21 ± 13.77	62.96 ± 13.42	< 0.0001
Age < 65	1852 (37.8%)	739 (56.6%)	< 0.0001
Age > = 65	3045 (62.2%)	567 (43.4%)	
Male	3283 (67.0%)	1023 (78.3%)	< 0.0001
Risk factors			
Dyslipidemia	1681 (34.3%)	466 (35.7%)	0.3607
Hypertension	2974 (60.7%)	705 (54.0%)	< 0.0001
Diabetes mellitus	2550 (52.1%)	562 (43.0%)	< 0.0001
Cardiovascular history			
Coronary artery disease	2190 (44.7%)	495 (37.9%)	< 0.0001
Prior myocardial infarction	783 (16.0%)	194 (14.9%)	0.3171
Heart failure	950 (19.4%)	150 (11.5%)	< 0.0001
Atrial fibrillation	253 (5.2%)	26 (2.0%)	< 0.0001
Peripheral vascular disease	109 (2.2%)	20 (1.5%)	0.1181
Comorbidity			
Renal dysfunction	1064 (21.7%)	145 (11.1%)	< 0.0001
Peptic ulcers	912 (18.6%)	180 (13.8%)	< 0.0001
Ischemic stroke	765 (15.6%)	121 (9.3%)	< 0.0001
Intracerebral hemorrhage	68 (1.4%)	9 (0.7%)	0.0425
Cancer	616 (12.6%)	154 (11.8%)	0.4432
Chronic obstructive pulmonary disease	933 (19.1%)	176 (13.5%)	< 0.0001
In-hospital procedure or surgery			
Intra-aortic balloon pump	133 (2.7%)	47 (3.6%)	0.0913
Revascularization	3397 (69.4%)	1105 (84.6%)	< 0.0001
Percutaneous coronary intervention	3231 (66.0%)	1102 (84.4%)	< 0.0001
Coronary artery bypass graft	183 (3.7%)	3 (0.2%)	< 0.0001
In-hospital drug use			
Beta blocker	3196 (65.3%)	962 (73.7%)	< 0.0001
ACEI or ARB	3255 (66.5%)	1011 (77.4%)	< 0.0001
Statin	3299 (67.4%)	1083 (82.9%)	< 0.0001
Calcium channel blocker	1939 (39.6%)	415 (31.8%)	< 0.0001
PPI	1126 (23.0%)	170 (13.0%)	< 0.0001
GP IIb/IIa inhibitor	318 (6.5%)	58 (4.4%)	0.0057

Data given as mean ± SD or n (%).

*ACEI* angiotensin-converting enzyme inhibitor, *ARB* angiotensin receptor blocker, *GP* glycoprotein.

**Table 2:** Baseline characteristics of subjects with non-ST elevation myocardial infarction after propensity score matching.

	Clopidogrel	Ticagrelor	*p*-value
	(N = 1303)	(N = 1303)	
Age (mean ± SD)	63.00 ± 14.23	62.99 ± 13.42	0.991
Age < 65	738 (56.6%)	736 (56.5%)	0.937
Age > = 65	565 (43.4%)	567 (43.5%)	
Male	1030 (79.0%)	1020 (78.3%)	0.633
Risk factors			
Dyslipidemia	464 (35.6%)	464 (35.6%)	1.000
Hypertension	685 (52.6%)	703 (54.0%)	0.480
Diabetes mellitus	566 (43.4%)	561 (43.1%)	0.843
Cardiovascular history			
Coronary artery disease	496 (38.1%)	494 (37.9%)	0.936
Prior myocardial infarction	201 (15.4%)	193 (14.8%)	0.662
Heart failure	155 (11.9%)	150 (11.5%)	0.761
Atrial fibrillation	32 (2.5%)	26 (2.0%)	0.426
Peripheral vascular disease	17 (1.3%)	20 (1.5%)	0.619
Comorbidity			
Renal dysfunction	140 (10.7%)	145 (11.1%)	0.754
Peptic ulcers	185 (14.2%)	179 (13.7%)	0.735
Ischemic stroke	131 (10.1%)	121 (9.3%)	0.508
Intracerebral hemorrhage	7 (0.5%)	9 (0.7%)	0.616
Cancer	168 (12.9%)	153 (11.7%)	0.371
Chronic obstructive pulmonary disease	168 (12.9%)	176 (13.5%)	0.643
In-hospital procedure or surgery			
Intra-aortic balloon pump	47 (3.6%)	44 (3.4%)	0.749
Revascularization	1112 (85.3%)	1102 (84.6%)	0.584
Percutaneous coronary intervention	1109 (85.1%)	1099 (84.3%)	0.586
Coronary artery bypass graft	5 (0.4%)	3 (0.2%)	0.479
In-hospital drug use			
Beta blocker	959 (73.6%)	959 (73.6%)	1.000
ACEI or ARB	1011 (77.6%)	1008 (77.4%)	0.888
Statin	1070 (82.1%)	1080 (82.9%)	0.606
Calcium channel blocker	398 (30.5%)	414 (31.8%)	0.499
PPI	172 (13.2%)	170 (13.0%)	0.908
GP IIb/IIa inhibitor	56 (4.3%)	57 (4.4%)	0.923

Data given as mean ± SD or n (%).

*ACEI* angiotensin-converting enzyme inhibitor, *ARB* angiotensin receptor blocker, *GP* glycoprotein.

### Clinical outcomes

The median (interquartile range, IQR) follow-up period of primary efficacy and safety endpoints were 5.2 (2.3–8.5) months. Before propensity score matching, the incidence of primary efficacy endpoint (death from any cause/non-fatal MI/non-fatal stroke) was 20.2% in the ticagrelor users and 35.4% in the clopidogrel users (adjusted HR 0.87; 95% CI 0.71–1.07). The incidence of primary safety endpoint (bleeding requiring hospitalization/major GI bleeding and/or intracerebral hemorrhage) was 2.2% in the ticagrelor users and 3.8% in the clopidogrel users (adjusted HR 0.72; 95% CI 0.39–1.30)(Table S1).

**Figure 2:**
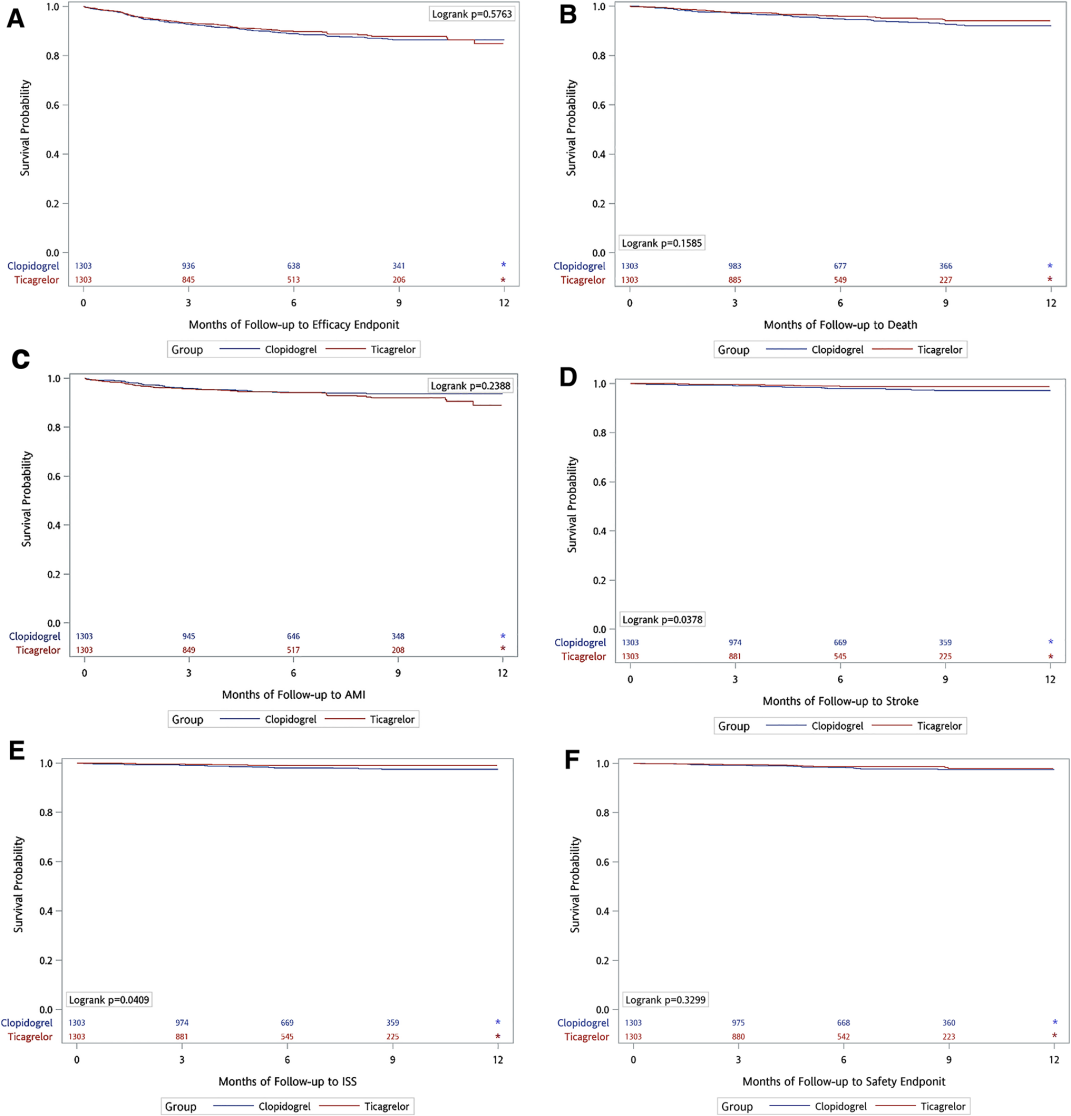
(**A**,**B**,**C**,**D**,**F**) *According to the regulation of NHIRD, data could not be present if the numbers of event were < 3. (**E**) ISS: Ischemic Stroke.

After propensity-score matching (Table 3), the incidence of primary efficacy endpoint was 20.3% in the ticagrelor users and 20.7% in the clopidogrel users (adjusted HR 0.94; 95% CI 0.73–1.22; Fig. 2A). The incidence of primary safety endpoint was 2.3% in the ticagrelor users and 3.2% in the clopidogrel users (adjusted HR 0.67; 95% CI 0.33–1.35; Fig. 2F). Regarding the secondary efficacy endpoints, patients in the ticagrelor group had significantly lower incidence of total stroke and ischemic stroke than clopidogrel users (total stroke: adjusted HR 0.44; 95% CI 0.21–0.94; *p* = 0.033; Fig. 2D; ischemic stroke: adjusted HR 0.42; 95% CI 0.19–0.94; *p* = 0.034; Fig. 2E).

**Table 3:** Efficacy and safety outcomes of NSTEMI after propensity score matching.

	After PS-Matched					
	Clopidogrel		Ticagrelor		Adjusted HR (95%CI) (versus Clopidogrel)	*p*-value
	Event number	Incident Rate (per person-year) (%)	Event number	Incident Rate (per person-year) (%)		
Primary efficacy endpoint	133	20.7	112	20.3	0.94 (0.73–1.22)	0.651
All-cause mortality	67	10.0	45	7.8	0.80 (0.54–1.18)	0.263
Non-fatal MI	65	10.0	72	13.0	1.25 (0.89–1.75)	0.197
Stroke	24	3.6	10	1.7	0.44 (0.21–0.94)	0.033
Ischemic stroke	22	3.3	9	1.6	0.42 (0.19–0.94)	0.034
Primary safety endpoint	21	3.2	13	2.3	0.67 (0.33–1.35)	0.259
Major GI bleeding	19	2.9	12	2.1	0.68 (0.32–1.43)	0.307

### Sensitivity analysis

Based on the use of P2Y12 inhibitors during hospitalization and after discharge, we categorized both the ticagrelor and clopidogrel groups into “ticagrelor without switching” subgroup (N = 940), “from none/clopidogrel switching to ticagrelor” subgroup (N = 363), “clopidogrel without switching” subgroup (N = 1096), and “from none/ticagrelor switching to clopidogrel” subgroup (N = 207) (Table 4). The primary efficacy and safety endpoints were comparable between “ticagrelor without switching” and "clopidogrel without switching” subgroups. The incidence of total stroke and ischemic stroke remained significantly lower in “ticagrelor without switching” subgroup compared to “clopidogrel without switching” subgroup.

**Table 4:** Subgroup analysis of the effect of drug switching strategies on the occurrence of the primary efficacy and safety endpoints.

	Primary efficacy endpoint		All-cause mortality		AMI		Stroke		Ischemic Stroke	
	Adjusted HR (95%CI)	*p*-value	Adjusted HR (95%CI)	*p*-value	Adjusted HR (95%CI)	*p*-value	Adjusted HR (95%CI)	*p*-value	Adjusted HR (95%CI)	*p*-value
Clopidogrel without switching	1		1		1		1			
Ticagrelor without switching	0.96 (0.71–1.29)	0.770	0.71 (0.45–1.12)	0.145	1.36 (0.90–2.07)	0.146	0.41 (0.17–0.98)	0.044	0.38 (0.15–0.97)	0.043
From none/ticagrelor switching to clopidogrel	1.68 (1.10–2.57)	0.017	0.92 (0.45–1.89)	0.817	2.62 (1.53–4.48)	0.000	0.57 (0.13–2.49)	0.453	0.68 (0.15–3.00)	0.609
From none/clopidogrel switching to ticagrelor	1.23 (0.84–1.80)	0.283	1.00 (0.56–1.76)	0.989	2.02 (1.24–3.28)	0.005	0.43 (0.12–1.46)	0.176	0.47 (0.14–1.63)	0.233

	Primary safety endpoint		Major GI bleeding							
	Adjusted HR (95%CI)	*p*-value	Adjusted HR (95%CI)	*p*-value						
Clopidogrel without switching	1		1							
Ticagrelor without switching	0.57 (0.25–1.34)	0.197	0.52 (0.21–1.28)	0.153						
From none/ticagrelor switching to clopidogrel	0.90 (0.25–3.18)	0.865	0.61 (0.13–2.78)	0.520						
From none/clopidogrel switching to ticagrelor	0.86 (0.31–2.39)	0.770	0.94 (0.33–2.66)	0.909						

Both “from none/ticagrelor switching to clopidogrel” and “from none/clopidogrel switching to ticagrelor” subgroups had significant higher incidence of non-fatal MI compared to patients continuously on clopidogrel (adjusted HR 2.62; 95% CI 1.53–4.48; adjusted HR 2.02; 95% CI 1.24–3.28, respectively).

## Discussion

This nationwide population-based, real-world study was conducted to compare the efficacy and safety between ticagrelor and clopidogrel in Taiwanese patients with NSTEMI. The main findings are threefold. First, patients treated with ticagrelor had similar ischemic vascular events and major bleeding risk as patients treated with clopidogrel. There were no safety concerns with ticagrelor as observed in the PHILO and TICAKOREA trials. Second, ticagrelor was associated with a significantly lower risk of ischemic stroke compared with clopidogrel in patients with NSTEMI. This finding, albeit needing verification, is of significance given that Asian populations have a predilection for stroke. Finally, the incidence of primary efficacy endpoint remained high, around 20% within the first year following NSTEMI. This finding suggests that guideline-directed therapy and long-term adherence should be regularly monitored and managed, even in countries with universal health coverage like Taiwan.

Ticagrelor, a direct-acting antiplatelet agent, inhibits the P2Y12 receptor in platelets without additional metabolic activation. The effects of total stroke and ischemic stroke reduction are consistent with the results of a recent, international, randomized THALES trial (Acute Stroke or Transient Ischemic Attack Treated with Ticagrelor and ASA for the Prevention of Stroke and Death), which was published in the New England Journal of Medicine^12^. Patients with mild-to-moderate acute ischemic stroke or high-risk transient ischemic attack (TIA), who were assigned within 24 h of symptom onset to receive 30-day treatment with Ticagrelor–aspirin, had a lower risk of stroke or death at 30 days than those who were assigned to receive aspirin alone. A benefit was observed with Ticagrelor–aspirin with respect to the incidence of the secondary outcome of subsequent ischemic stroke, which was lower than that with aspirin alone; however, a benefit was not observed with respect to the incidence of overall disability, and the incidence of severe hemorrhage was higher among patients who received ticagrelor–aspirin than among those who received aspirin alone during a 30-day treatment period. Similar results were also observed in the Platelet-Oriented Inhibition in New TIA and Minor Ischemic Stroke (POINT) trial, which compared clopidogrel–aspirin with aspirin alone in patients with acute minor stroke and TIA^13^. There was no specific information regarding direct comparison between clopidogrel–aspirin and ticagrelor-aspirin for minor stroke/TIA management, and our study provided a hypothesis-generating finding in this field among patients with NSTEMI events.

Lee CH et al. recently reported results of nationwide claim-based cohort study regarding the efficacy and safety outcomes of Taiwanese patients with acute MI treated with ticagrelor or clopidogrel. It showed that ticagrelor use reduced the rate of death from any cause, MI, or stroke without an increase in overall major bleeding during an 18-month follow-up^14^. There was a trend toward lower total stroke (adjusted HR 0.745, 95% CI 0.542–1.023) and ischemic stroke rates (adjusted HR 0.684, 95% CI 0.457–1.023) in patients with acute MI treated with ticagrelor. In our study, we did not observe that ticagrelor user was associated with a reduction in the primary efficacy endpoint in patients with NSTEMI. Nevertheless, a statistically significant reduction in stroke was noted in patients treated with ticagrelor.

The importance of stroke has not been fully evaluated in randomized trials since those studies excluded patients with severe heart failure or rarely included patients with Killip class > II^2^ who would be at higher risk for stroke^15, 16^. According to previous studies, Asians had higher risk for stroke after acute coronary syndrome than people of white ethnicity^16, 17^. A PLATO sub-study demonstrated a higher incidence of stroke in Asian (2.0%) than in non-Asian patients (1.2%), and a non-significant reduction of stroke by ticagrelor among East Asian patients (clopidogrel vs. ticagrelor, 1.5% vs. 0.7%)^18^. It is reasonable to assume that the potential risk of stroke following NSTEMI could be more pronounced in Asian patients. The finding that use of ticagrelor was associated with a lower incidence of stroke is of clinical significance in this regard.

Patients seen in real-world may differ in several aspects from those in clinical trials, including more complicated comorbidities and lower adherence to medications^7^. This may be one of the reasons why the incidence of primary efficacy endpoint in the present study was higher than that in the NSTEMI subgroup of the PLATO trial^19^. In the NSTEMI subgroup (n = 11,080) of PLATO trial, the primary composite efficacy endpoint (death from cardiovascular causes, myocardial infarction or stroke) was 10.0% in ticagrelor group and 12.3% in clopidogrel users. Although recent guidelines suggest early invasive management in NSTEMI^1, 20, 21^, nearly half of NSTEMI patients in the PLATO trial were treated with optimal medical therapy alone. Patients who are managed noninvasively usually have more co-morbidities, uncertain bleeding risk, and unpredictable outcomes than patients who are revascularized^22^. Compared with landmark clinical trials^19, 23^, a higher PCI (69.9%) rate was noted in our database which reflected the real-world practice in the PCI era. However, the higher PCI rate did not translate into better outcomes within the first year following discharge from NSTEMI.

The PHILO trial, which was designed to mirror PLATO, was a multicenter, randomized, double-blind, non-event-driven study conducted in Japan and East Asian countries (Japan, 90%; Taiwan, 4%). At 12 months, overall major bleeding occurred in 10.3% of ticagrelor-treated patients and in 6.8% of clopidogrel-treated patients (hazard ratio 1.54, 95% CI 0.94–2.53)^5^. The overall incidence of bleeding in our study was lower than that in the PHILO and PLATO trials. These can be attributed in part to different patient characteristics, including ethnicity, and possibly suboptimal drug adherence. Another reason is that bleeding events not requiring hospitalization might not be adequately documented in the Taiwan National Health Insurance Database. Compared to Western populations, East Asian people were found to be more susceptible to bleeding complications and had relatively lower thromboembolic events^24–27^. Both PHILO and TICAKOREA trials and results from the Korea Acute Myocardial Infarction Registry—National Institute of Health showed that, compared to clopidogrel, ticagrelor did not further reduce ischemic events, and was associated with increased risk of bleeding complications^6, 28^. However, all these Asian populations-based studies are not specifically designed for NSTEMI patients. In the present study, Taiwanese patients with NSTEMI treated with ticagrelor, as compared to clopidogrel, had similar rates of primary efficacy endpoint and overall major bleeding. In contrast to prior Asian population-based studies, we herein did not observe a safety signal of ticagrelor.

The results of similar primary efficacy and safety endpoints between ticagrelor and clopidogrel remained irrespective of whether the P2Y12 inhibitors used during admission were changed while during follow-up^29^. It has been shown that switching from one anti-platelet agent to another occurred mostly after discharge, and the most frequent cause was the need of oral anticoagulation treatment, followed by bleeding events^29–31^. Our analysis intriguingly found that the incidence of non-fatal MI increased significantly if switching of P2Y12 inhibitors occurred in either direction. More studies are needed to validate this finding.

There are several limitations in the present study. First, this is a retrospective cohort study, not a randomized controlled study. Therefore, selection bias was hardly avoidable, even though both propensity-score matching and sensitivity analyses were performed. Second, we could not accurately evaluate the minor bleeding complications because these events were possibly unrecorded and incomplete in the Taiwan National Health Insurance Database. Detailed information on the causes of bleeding including vascular access complications was also not available. Third, the health-care claims data did not contain body mass index, coronary artery disease severity, revascularization details, and smoking status. We could not adjust for the above-mentioned factors. Fourth, we did not include in-hospital events, and medication adherence during follow-up was not available. Fifth, the median follow-up time in the study was only 5.2 months and the finding of stroke reduction with ticagrelor compared to clopidogrel needs further verification in the near future.

## Conclusion

In this nationwide population-based study of East Asian patients with NSTEMI, outpatient treatment with aspirin and ticagrelor, as compared to aspirin and clopidogrel, was associated with similar rates of ischemic composite outcomes and overall major bleeding. The reductions in total stroke and ischemic stroke with ticagrelor use, a hypothesis-generating finding, merits further verification.

## Supplementary Information

Supplementary Information.

## Data Availability

The data that support the findings of this study are available from Taiwan National Health Insurance Research Database but restrictions apply to the availability of these data, which were used under license for the current study, and so are not publicly available. Data are however available from the authors upon reasonable request and with permission of Taiwan National Health Insurance Research Database.
