# Supplementary material for: Efficacy and safety of ticagrelor versus clopidogrel in patients with non-ST-elevation myocardial infarction in Taiwan
Source: Sci Rep. 2021 Jul 8;11:14150. doi: 10.1038/s41598-021-93712-9 (PMC8266799; doi:10.1038/s41598-021-93712-9)
Supplement: Supplementary file 1 — Supplementary Information. [file 41598_2021_93712_MOESM1_ESM.docx]

**Table S1. Before Matching**

|  | ***before PS-Matched*** | | | |  |  |  |
| --- | --- | --- | --- | --- | --- | --- | --- |
|  | **Clopidogrel** | | **Ticagrelor** | |  | **adjusted HR (95%CI)** | ***p*-value** |
|  | **Event  Number** | **Incident Rate** (per person-year) | **Event  Number** | **Incident Rate** (per person-year) |  |  |  |
| **Primary efficacy endpoint** | 827 | 35.4% | 112 | 20.2% |  | 0.87 (0.71-1.07) | 0.185 |
| All-cause mortality | 474 | 19.0% | 45 | 7.7% |  | 0.75 (0.55-1.02) | 0.069 |
| Non-fatal MI | 385 | 16.3% | 72 | 12.9% |  | 1.03 (0.80-1.34) | 0.803 |
| Stroke | 111 | 4.5% | 10 | 1.7% |  | 0.55 (0.29-1.07) | 0.078 |
| Ischemic stroke | 100 | 4.1% | 9 | 1.6% |  | 0.56 (0.28-1.13) | 0.107 |
| **Primary safety endpoint** | 95 | 3.8% | 13 | 2.2% |  | 0.72 (0.39-1.30) | 0.272 |
| Major GI bleeding | 85 | 3.4% | 12 | 2.1% |  | 0.72 (0.39-1.34) | 0.303 |
